# A systematic review of *in utero* cannabis exposure and risk for structural birth defects

**DOI:** 10.3389/fped.2023.1149401

**Published:** 2023-05-25

**Authors:** Ayesha C. Sujan, Anish Pal, Lyndsay A. Avalos, Kelly C. Young-Wolff

**Affiliations:** ^1^ Department of Anesthesiology, Perioperative and Pain Medicine, Stanford University School of Medicine, Stanford, CA, United States; ^2^ Division of Research, Kaiser Permanente Northern California, Oakland, CA, United States; ^3^ Herbert Wertheim College of Medicine, Florida International University, Miami, FL, United States; ^4^ Department of Psychiatry and Behavioral Sciences, University of California, San Francisco, CA, United States

**Keywords:** pregnancy, prenatal exposure, *in utero* exposure, cannabis, marijuana

## Abstract

**Introduction:**

Cannabis use among pregnant women has increased over time. Therefore, there is a great public health need to understand the consequences of *in utero* cannabis exposure. While several meta-analyses and reviews have summarized the evidence of *in utero* cannabis exposure on adverse obstetric outcomes (e.g., low birth weight and preterm birth) and long-term offspring development, there has not been a focus on *in utero* cannabis exposure and risk for structural birth defects.

**Methods:**

We conducted a systematic review using PRISMA guidelines to evaluate the association between *in utero* cannabis exposure and structural birth defects.

**Results:**

We identified 20 articles to include in our review and focused on interpreting findings from the 12 that adjusted for potential confounders. We report findings by seven organ systems. Within the 12 articles, four reported on cardiac malformations, three reported on central nervous system malformations, one reported on eye malformations, three reported on gastrointestinal malformations, one reported on genitourinary malformations, one reported on musculoskeletal malformations, and two reported on orofacial malformations.

**Discussion:**

Findings on associations between *in utero* cannabis exposure and birth defects reported in more than two articles were mixed (i.e., findings for cardiac, gastrointestinal, central nervous system malformations). Findings for associations between *in utero* cannabis exposure and birth defects reported in two articles (i.e., orofacial malformations) or in a single article (eye, genitourinary, and musculoskeletal) suggested that cannabis exposure was not associated with these types of malformations, but strong conclusions cannot be drawn from such sparce research. We review the limitations and gaps in the existing literature and call for more research to rigorously evaluate associations between *in utero* cannabis exposure and structural birth defects.

**Systematic Review Registration:**

identifier CRD42022308130.

## 1. Introduction

Research has documented an increase in rates of cannabis use among pregnant people over time. Among a nationally representative sample of pregnant individuals in the United States, the prevalence of self-reported prenatal cannabis use in the past month increased from 3.4% in 2002–2003, to 7.0% in 2016–2017 (1). Prenatal cannabis use may increase even more rapidly as more US states legalize cannabis for recreational use (2–7). Moreover, cannabis use in pregnancy could impact fetal development because cannabis is lipid soluble and is able to cross the placenta and blood-brain barrier (8), and some previous studies have suggested a potential link between in-utero cannabis exposure and adverse offspring outcomes [e.g., (9)]. Therefore, there is a great public health need to understand the consequences of *in utero* cannabis exposure on offspring development. Several meta-analyses and reviews have summarized the evidence of *in utero* cannabis exposure on adverse obstetric outcomes (e.g., low birth weight and preterm birth) and long-term offspring development (8, 10–15). However, reviews to date have not focused on research regarding *in utero* cannabis exposure and risk for structural birth defects. The causes and risk factors for many structural birth defects remains unknown, and understanding preventable causes and risk factors for structural birth defects is particularly important given the strong association between birth defects and morbidity/mortality (16). Given this need, we conducted a systematic review using Preferred Reporting Items for Systematic Reviews and Meta-Analyses (PRISMA) guidelines to evaluate whether *in utero* cannabis exposure is associated with structural birth defects compared to pregnancies with no cannabis exposure (Prospective Register of Systematic Reviews [PROSPERO] registration number: CRD42022308130; (17)].

## 2. Methods

Web of Science and PubMed databases were searched for English language articles published before February 1, 2022 utilizing the following key words: “(Pregnancy OR Prenatal OR *In utero* OR Perinatal) AND (Cannabis OR Marijuana) AND (Birth defects OR Congenital malformations OR Congenital anomalies OR Central nervous system defect OR Neural tube defects OR Holoprosencephaly OR Microcephaly OR Ear defect OR Eye defect OR Gastrointestinal defect OR Biliary atresia OR Esophageal atresia OR Tracheoesophageal fistula OR Intestinal atresia OR Intestinal stenosis OR Pyloric stenosis OR Hypospadias OR Renal agenesis OR Renal hypoplasia OR Renal dysplasia OR Cardiac defect OR Musculoskeletal defect OR Congenital diaphragmatic hernia OR Gastroschisis OR Limb deficiency OR Omphalocele OR Orofacial defect OR Respiratory defect OR Choanal atresia OR Cleft lip OR Cleft palate).” The inclusion criteria were English-language articles and epidemiological studies. Animal studies and review articles were excluded as the focus of our review was strictly on human outcomes.

**Figure 1:**
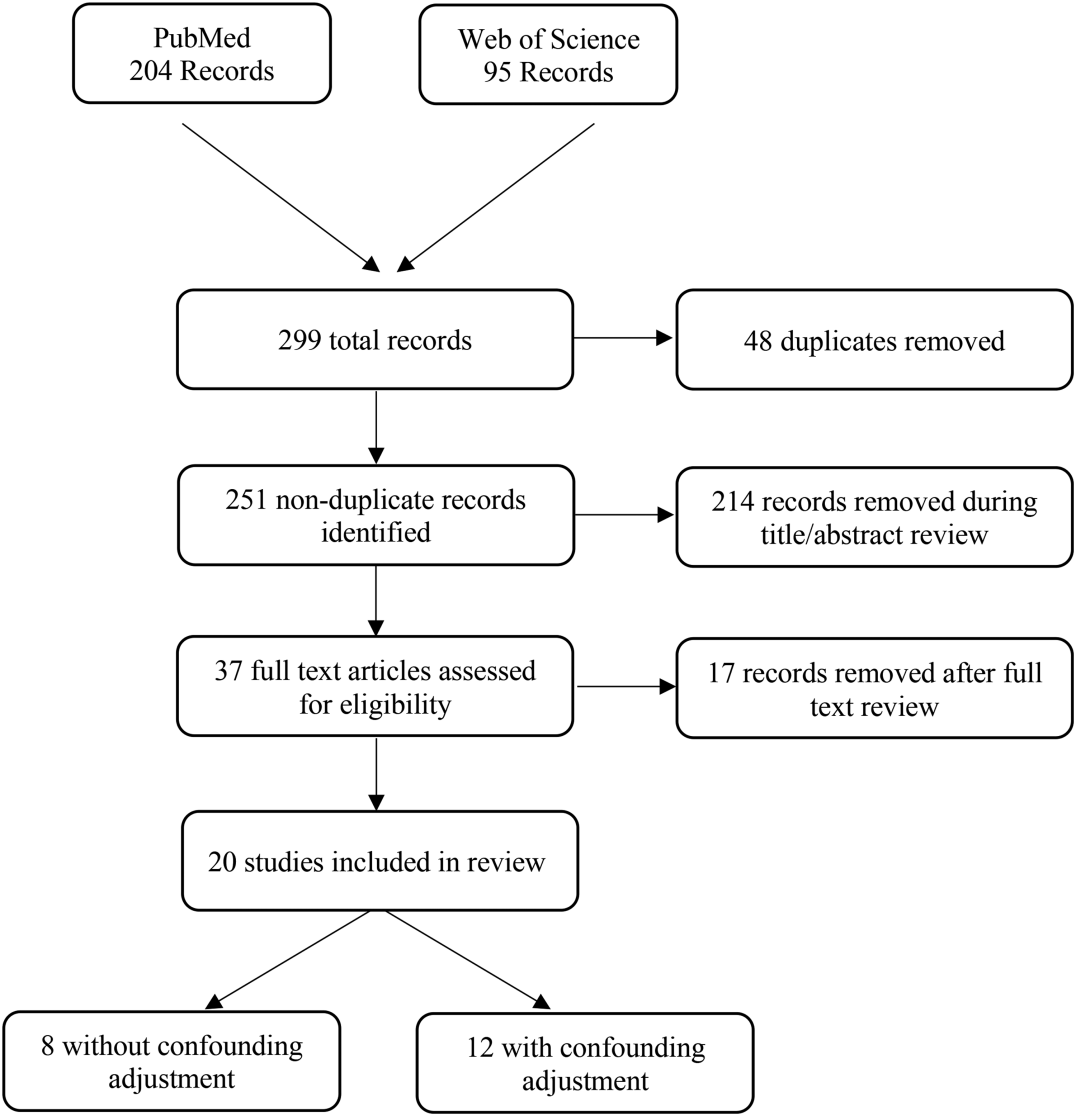
PRISMA flow diagram.

The search revealed 299 potentially relevant articles of which 48 were duplicates. We created an EndNote library of 251 non-duplicate articles. Two authors then independently reviewed the titles and abstracts of the articles in the EndNote library to exclude articles that did not meet the inclusion criteria. After their independent reviews, the two authors discussed disagreements and together decided to include 37 articles for a full text review. During the full text review, 17 additional articles were excluded for the following reasons: study design was a case study (18), a comparable study was conducted by the same authors using the same dataset (19–23), and the study did not specifically evaluate associations between cannabis exposure in pregnancy and birth defects [e.g., cannabis was included in a general substance use exposure variable or the outcome studied was not a birth defect; (24–34)]. Therefore, the final review included 20 articles (9, 22, 35–53). See Figure 1 for a PRISMA flow diagram illustrating our identification process of articles for our final review.

## 3. Results

### 3.1. Study characteristics

Of the 20 included articles, 8 were from prospective studies using recruited samples (35–42), and 12 were from retrospective cohort or case-control studies using health care records (9, 43–53). Samples sizes varied from 50 to 3,067,069. Earliest birth years for cohorts varied from 1968 to 1980. Only 3 of the articles reported on studies using urine toxicology tests (46–48); the rest reported on studies that relied on self-report to measure prenatal cannabis use. Of the 17 articles that reported on studies using self-reports to measure cannabis use, 16 had measures of self-reported cannabis use, and 1 had a measure of self-reported cannabis-related diagnoses (43). The outcome definitions varied across studies with some investigating associations with specific malformations and other studies investigating associations with any malformation. While 8 studies did not adjust for any potential confounders (38, 40, 41, 44, 46, 47, 49, 53), the rest adjusted for confounding, though the specific factors adjusted for varied across studies. Table 1 provides information about the characteristics of each individual study.

**Table 1:** Description of 20 articles (listed in alphabetical order) included in the final review.

Citation	Design and sample	Sample size	Birth years	Cannabis exposure definition	Organ System	Birth defect	Confounding adjustment
1. Astley et al. (41)	Prospective study of patients in their sixth month of pregnancy recruited from a health maintenance organization in Seattle, Washington, USA	80 (40 exposed matched to 40 unexposed)	1982–1984	Self-reported use during the first trimester of pregnancy ascertained at an interview 6 weeks after delivery	Orofacial	Fetal alcohol like facial characteristics	None
2. Bandoli et al. (43)	Retrospective, population-based cohort of births in California, USA	3,067,069	2011–2017	Self-reported cannabis-related diagnoses made during pregnancy or delivery	Cardiac, Central nervous system, Eye, Gastrointestinal, Orofacial	Any cardiac malformation, Any central nervous malformation, Neural tube defect Anencephaly, Spina Bifida, Eye malformation Gastrointestinal malformation	Demographics: race and ethnicity, payer source, maternal age and education Substance use: alcohol abuse, and nicotine and substance-related diagnoses Mental health: anxiety, depression, bipolar disorder Physical health: pre-pregnancy body mass index (BMI), preexisting hypertension, preexisting diabetes
3. Bourque et al. (44)	Retrospective, population-based cohort study of births in Ontario, Canada	1,001,080	2012–2018	Self-reported use during pregnancy ascertained at the first prenatal visit or admission for birth	Gastrointestinal	Gastroschisis	None
4. Coleman-Cowger et al. (35)	Prospective study of patients recruited from two obstetric clinics in Maryland, USA	338	2017	Self-reported use in the last month ascertained at prenatal visits	Unspecified	Birth defects	Demographics: marital status Pregnancy-specific: trimester of self-reported use
5. Cornelius et al. (36)	Prospective study of patients 18 years or younger recruited from an outpatient prenatal clinic in Pittsburgh, USA	310	1990–1993	Self-reported first-trimester use	Unspecified	Major and minor physical anomalies	Demographics: race, infant sex, maternal age, household structure, mother's parent's education, full-time or part-time school status, Substance use: alcohol use, marijuana, cocaine/crack, and other illicit drug use Mental health: social support, depression Physical health: pre-pregnancy weight, gestational weight gain, maternal height, maternal nutrition Pregnancy-specific: gestational age at birth, gravidity, adequacy of prenatal care
6. Day et al. (37)	Prospective study of patients recruited from an outpatient prenatal clinic in Pittsburgh, USA	763	1982–1985	Self-reported use at the fourth prenatal month visit, seventh prenatal month visit, and postpartum hospital stay about use in each trimester	Unspecified	Minor and major physical abnormalities	Demographics: maternal age, education, marital status, work status, income, race, Substance use: use of tobacco, alcohol, and other illicit drugs Mental health: social support, depression and anxiety Physical health: gestational weight gain, maternal height Pregnancy-specific: gravidity Other: life events
7. Downing et al. (45)	Retrospective, population-based, case-control study of records from 10 Centers for Birth Defects Research and Prevention across the USA	11,964 (135 cases, 11,829 controls)	1997–2011	Self-report of use during the first trimester of pregnancy ascertained between 6 and 24 months after delivery	Cardiac	Ebstein anomaly	Demographics: maternal age at delivery, paternal age at delivery, birth year, maternal race/ethnicity Substance use: none Mental health: none Physical health: maternal pre-pregnancy body mass index Pregnancy-specific: season of conception Other: family history of congenital heart defects
8. Forrester et al. (46)	Retrospective, population-based, case-control study of births in Hawaii, USA	316,508	1986–2002	Urine toxicology during or shortly after delivery OR report of use on medical record	Gastrointestinal	Gastroschisis	None
9. Forrester et al. (47)	Retrospective, population-based, case-control study of births in Hawaii, USA	316,508	1986–2002	Urine toxicology during or shortly after delivery OR report of use on medical record	Cardiac, Central nervous system, Eye, Gastrointestinal, Genitourinary, Musculoskeletal, Orofacial	54 selected birth defects (see paper)	None
10. Gibson et al. (42)	Prospective study of patients recruited from a hospital in London, England	7,301	1975–1981	Self-report use up to once a week and more than once a week at antenatal interview	Unspecified	Congenital anomalies	Demographics: maternal age Substance use: alcohol use, tobacco use Mental health: none Physical health: none Pregnancy-specific: parity Other: none
11. Hingson et al. (38)	Prospective study of patients recruited from a hospital in Boston, USA	1,690	1977–1979	Self-report use during pregnancy ascertained post delivery	Orofacial	Features compatible with fetal alcohol syndrome	None
12. Kharbanda et al. (48)	Retrospective, cohort study of births in Minnesota, USA	3,435	2015–2017	Urine toxicology screens at the first prenatal visit (generally between 6 and 14 weeks)	Unspecified	Major structural birth defect	Demographics: maternal race/ethnicity, age, Substance use: smoking during pregnancy Mental health: Physical health: pre-pregnancy body mass index Pregnancy-specific: none Other: none
13. Lam et al. (49)	Retrospective, case-control of births in California, USA	149 (55 cases, 94 control)	1988–1990	Self-reported when infant 3–6 months old	Gastrointestinal	Gastroschisis	None
14. Linn et al. (39)	Prospective study of patients recruited from a hospital in Boston, USA	12,424	1977–1980	Self-reported use during pregnancy ascertained during delivery admission	Unspecified	Major or minor malformations	Demographics: race, maternal age 35 or older, on welfare Substance use: alcohol use in pregnancy, smoking 3 or more cigarettes per day at delivery Mental health: none Physical health: previous miscarriages, previous stillbirths, previous induced abortions Pregnancy-specific: parity greater than 1 Other: none
15. O’Connell et al. (40)	Prospective study of patients recruited from a hospital in Ottawa, Canada	50	Exact dates unknown (recruitment in 1978)	Self-reported use during pregnancy	Orofacial	Any minor physical anomalies, Anomalies of face and head	None
16. Shaw et al. (50)	Retrospective, population-based, case-control study of births in California, USA	1,077 (538 cases, 539 controls)	1989–1991	Self-reported use 3 months before pregnancy through pregnancy	Central Nervous System	Neural tube defect	Demographics: race/ethnicity, education, income, age Substance use: use of other substances in the periconception period Mental health: none Physical health: maternal vitamin use Pregnancy-specific: none Other: none
17. Torfs et al. (51)	Retrospective, population-based case-control study of births in California, USA	330 (110 cases, 220 controls)	1988–1990	Self-reported first-trimester use	Gastrointestinal	Gastroschisis	Demographics: maternal age Substance use: none Mental health: none Physical health: none Pregnancy-specific: none Other: none
18. Van Gelder et al. (52)	Retrospective, case-control study of births in 10 states that were part of the National Birth Defects Study^a^	20,415 (13,859 cases, 6,556 controls)	1997–2005	Self-reported use in the month before pregnancy or during the first 3 months of pregnancy	Cardiac, Gastrointestinal, Genitourinary, Musculoskeletal, Orofacial	Atrial septal defect not otherwise specified, Atrial septal defect secundum, Coarctation of Aorta, Dextrotransposition of the great arteries, Hypoplastic left heart syndrome, Peri membranous ventricular septal defect, Pulmonary valve stenosis, Tetralogy of Fallot, Anorectal atresia, Diaphragmatic hernia, Esophageal atresia with/without tracheoesophageal fistula, Gastroschisis, Hypospadias, Craniosynostosis, Transverse limb deficiency, Anotia/microtia, Cleft lip with or without cleft palate, Cleft palate	Demographics: maternal age at delivery, race or ethnicity, level of education Substance use: smoking in the periconceptional period, binge drinking in the periconceptional period Mental health: none Physical health: pre-pregnancy body mass index, any periconceptional folic acid use Pregnancy-specific: none Other: none
19. Williams et al. (9)	Retrospective, case-control study of births in Atlanta, Georgia, USA	3,151 (122 cases, 3,029 controls)	1968–1980	Maternal and paternal self-reported frequency of use 3 months prior to pregnancy through the first trimester	Cardiac	Ventral septal defect	Demographics: maternal age, maternal race, infant race, birth period, and hospital of birth Substance use: none Mental health: Physical health: maternal diabetes, multivitamin use Pregnancy-specific: none Other: none
20. Witter et al. (53)	Retrospective study of patients in Baltimore, Maryland, USA	8,350	1983–1985	Self-reported use in pregnancy	Unspecified	Anomalies	None

^a^ The 10 states included in the study conducted by Van Gelder et al. (52) were Arkansas, California, Georgia, Iowa, Massachusetts, New Jersey, New York, North Carolina, Texas, and Utah.

### 3.2. Adjusted associations with specific birth defects

Table 2 includes information on adjusted associations between *in utero* cannabis exposure and specific birth defects. When examining associations, we only considered the 12 studies that adjusted for confounding, given the importance in doing so in assessing epidemiologic relationships (54). We included information about associations with specific malformations whenever available. However, given the rarity of specific malformations, most studies evaluated associations with organ specific malformations grouped together.

**Table 2:** Adjusted associations for specific birth defect, organized by organ system from 12 articles that adjust for confounding.

Organ system	Citation	Cannabis exposure definition	Birth defect	Association
Cardiac	Bandoli et al. (43)	Cannabis-related diagnosis made during pregnancy or delivery	Any cardiac malformation	RR: 1.0, 95% CI: 0.8–1.2
		Cannabis-related diagnosis without another substance use disorder diagnoses made during pregnancy or delivery		RR: 1.0, 95% CI: 0.8–1.3.
	Downing et al. (45)	Self-reported first-trimester use	Ebstein anomaly	OR: 1.8, 95% CI: 0.9–3.8
	Van Gelder et al. (52)	Self-reported use in the month before pregnancy or during the first trimester	Atrial septal defect not otherwise specified	OR: 1.1, 95% CI: 0.7–1.8
			Atrial septal defect secundum	OR: 0.8, 95% CI: 0.6–1.1
			Coarctation of Aorta	OR: 1.2, 95% CI: 0.7–1.5
			Dextrotransposition of the great arteries	OR: 0.8, 95% CI: 0.5–1.5
			Hypoplastic left heart syndrome	OR: 0.8, 95% CI: 0.4–1.5
			Peri membranous ventricular septal defect	OR: 1.0, 95% CI: 0.8–1.4
			Pulmonary valve stenosis	OR: 1.0, 95% CI: 0.7–1.9
			Tetralogy of Fallot	OR: 1.1, 95% CI: 0.7–1.7
	Williams et al. (9)	Any self-reported use 3 months before pregnancy through the first trimester	Ventral septal defect	OR: 1.9, 95% CI: 1.3–2.8
		Self-reported use <2 days/week 3 months before pregnancy through the first trimester		OR: 2.20, 95% CI: 1.2–3.9
		Self-reported use >3 days/week 3 months before pregnancy through the first trimester		OR: 3.7, 95% CI: 1.6–9.0
		Paternal-reported use <2 days/week 3 months before pregnancy through the first trimester		OR: 1.5, 95% CI: 0.6–3.9
		Paternal-reported use >3 days/week 3 months before pregnancy through the first trimester		OR: 3.2, 95% CI: 0.61–10.71
Central nervous system	Bandoli et al. (43)	Cannabis-related diagnosis made during pregnancy or delivery	Any central nervous system malformation	RR: 1.2, 95% CI: 1.0–1.5.
		Cannabis-related diagnosis without another substance use disorder diagnosis made during pregnancy or delivery		RR: 1.2, 95% CI: 0.9–1.6
	Shaw et al. (50)	Self-reported use 3 months before pregnancy through pregnancy	Neural tube defect	OR: 0.7, 95% CI: 0.5–1.2
	Van Gelder et al. (52)	Self-reported use in the month before pregnancy or during the first trimester	Anencephaly	OR: 2.2, 95% CI: 1.3–3.7
			Spina Bifida	OR: 0.9, 95% CI: 0.6–1.4
Eye	Bandoli et al. (43)	Cannabis-related diagnosis made during pregnancy or delivery	Eye malformation	RR: 1.1, 95% CI: 0.7–1.7
		Cannabis-related diagnosis without another substance use disorder diagnoses made during pregnancy or delivery		RR: 1.2, 95% CI: 0.7–2.2
Gastrointestinal	Bandoli et al. (43)	Cannabis-related diagnosis made during pregnancy or delivery	Any gastrointestinal malformation	RR: 1.3, 95% CI: 1.1–1.5
		Cannabis-related diagnosis without another substance use disorder diagnoses made during pregnancy or delivery		RR: 1.3, 95% CI: 1.0–1.6
	Torfs et al. (51)	Self-report of first-trimester use	Gastroschisis	OR: 4.5, 95% CI 2.1–9.8
	Van Gelder et al. (52)	Self-reported use in the month before pregnancy or during the first trimester	Anorectal atresia	OR: 0.8, 95% CI: 0.5–1.3
			Diaphragmatic hernia	OR: 1.4, 95% CI: 0.9–2.2
			Esophageal atresia with/without tracheoesophageal fistula	OR: 1.4, 95% CI: 0.8–2.4
			Gastroschisis	OR: 1.2, 95% CI: 0.9–1.7
Genitourinary	Van Gelder et al. (52)	Self-reported use in the month before pregnancy or during the first trimester	Hypospadias	OR: 0.8, 95% CI: 0.5–1.2
				OR: 0.8, 95% CI: 0.5–1.2
Musculoskeletal	Van Gelder et al. (52)	Self-reported use in the month before pregnancy or during the first trimester	Craniosynostosis	OR: 0.8, 95% CI: 0.5–1.3
			Transverse limb deficiency	OR: 1.0, 95% CI: 0.6–1.7
Orofacial	Van Gelder et al. (52)	Self-reported use in the month before pregnancy or during the first trimester	Anotia/microtia	OR: 0.9, 95% CI: 0.5–1.7
			Cleft lip with or without cleft palate	OR: 1.0, 95% CI: 0.8–1.3
			Cleft palate	OR: 1.0, 95% CI: 0.7–1.5
	Bandoli et al. (43)	Cannabis-related diagnosis made during pregnancy or delivery	Oral cleft	RR: 1.1, 95% CI: 0.9–1.5.
		Cannabis-related diagnosis without another substance use disorder diagnoses made during pregnancy or delivery		RR: 1.1, 95% CI: 0.8–1.5.
Unspecified	Coleman-Cowger et al. (35)	Self-reported use during pregnancy	Any birth defects	OR: 1.2, 95% CI: 0.5–0.9.
	Cornelius et al. (36)	Self-reported first-trimester use	Minor physical anomalies	OR: 3.2, 95% CI: 1.0–10.2
	Day et al. (37)	Self-reported use by trimester	Minor and major physical abnormalities	No significant association (point estimate not reported)
	Gibson et al. 1983 (42)	Self-reported use by trimester	Congenital defects	No significant association (point estimate not reported)
	Kharbanda et al. (48)	Urine toxicology screens during first prenatal visit	Major structural birth defects	RR: 0.6 95% CI: 0.2–2.0
	Linn et al. (39)	Self-reported use during pregnancy	Major or minor malformations	OR: 1.4, 95% CI: 1.0–1.9

RR, relative risk; OR, odds ratio; CI, confidence interval.

#### 3.2.1. Cardiac

Results were inconsistent across the four articles reporting findings from studies assessing associations between *in utero* cannabis exposure and cardiac malformations (9, 43, 45, 52). One article (9) indicated a dose-response relationship between self-reported cannabis use three months before pregnancy through the first trimester and ventral septal defect [any use OR: 1.9, 95% CI: 1.3, 2.8; use <2 days/week OR: 2.20, 95% CI: 1.2, 3.9; use >3 days/week OR: 3.7, 95% CI: 1.6, 9.0; (9)]. Another study found increased odds of Ebstein anomaly associated with maternal self-reported first-trimester cannabis use, though the confidence interval around the estimate was wide and included the null [OR: 1.8, 95% CI: 0.9, 3.8; (45)]. Additionally, two other articles (43, 52) reported no elevated risk of any cardiac malformation among infants born to individuals with a cannabis-related diagnosis made during pregnancy or delivery [RR: 1.0, 95% CI: 0.8, 1.2; (43)] and no associations between maternal self-reported cannabis use in the month before pregnancy or during the first trimester of pregnancy and eight specific cardiac malformations [Table 2; (52)].

#### 3.2.2. Central nervous system

Three articles reported findings from studies assessing *in utero* cannabis exposure and central nervous system (CNS) malformations, and results were conflicting (43, 50, 52). Two studies focused on neural tube defects [NTD; (50, 52)]—Van Gelder et al. reported on two subtypes of NTD [anencephaly and spina bifida; (52)], while Shaw et al. focused on any NTD (50). Van Gelder et al. reported increased odds of anencephaly [odds ratio [OR]: 2.2, 95% CI: 1.3–3.7; (52)] but not spina bifida [OR: 0.9, 95% CI: 0.6–1.4; (52)] among infants born to individuals who self-reported cannabis use in the month before pregnancy or during the first trimester of pregnancy (52); and, Shaw et al. failed to find an association between self-reported cannabis use three months before pregnancy through pregnancy and any NTD [OR: 0.7, 95% CI: 0.5–1.2; (50)]. The third study (43) found an increased risk of any CNS malformations among infants born to individuals with a self-reported cannabis-related diagnosis [relative risk [RR]: 1.2, 95% CI: 1.0, 1.5; (43)].

#### 3.2.3. Eye

One article reported on the association between *in utero* cannabis exposure and eye malformation (43). The study failed to find an association between a cannabis-related diagnosis made during pregnancy or delivery and eye malformation [RR: 1.1, 95% CI: 0.7, 1.7; (43)].

#### 3.2.4. Gastrointestinal

Three articles reported findings from studies assessing associations between *in utero* cannabis exposure and gastrointestinal malformations (43, 51, 52). The findings from these three studies were mixed. Two articles suggested *in utero* cannabis exposure was associated with increased risk of gastrointestinal malformations. Specifically, one article (43) reported an association between cannabis-related diagnoses made during pregnancy or delivery and any gastrointestinal malformation [RR: 1.3, 95% CI: 1.1, 1.5; (43)]; and, one article (51) reported an association between self-reported cannabis use in the first trimester and gastroschisis [OR: 4.5, 95% CI 2.1–9.8; (51)]. However, another article (52) did not find any significant associations between self-reported cannabis use in the month before pregnancy or during the first trimester and several specific gastrointestinal birth defects [Table 2; (51)], including gastroschisis [OR: 1.2, 95% CI: 0.9, 1.7 (52)].

#### 3.2.5. Genitourinary

One article reported on associations between *in utero* cannabis exposure and genitourinary malformations (52). This study failed to find an association between self-reported cannabis use in the month before pregnancy or during the first trimester and hypospadias [OR: 0.8, 95% CI: 0.5–1.2; (52)].

#### 3.2.6. Musculoskeletal

One article reported on association between *in utero* cannabis exposure and musculoskeletal malformations (52). The study failed to find associations between self-reported cannabis use in the month before pregnancy or during the first trimester and (a) craniosynostosis (OR: 0.8, 95% CI: 0.5–1.3) or (b) transverse limb deficiency [OR: 1.0, 95% CI: 0.6–1.7; (52)].

#### 3.2.7. Orofacial

Associations between *in utero* cannabis exposure and specific orofacial malformations were reported on in two articles (43, 52). Both articles reported associations close to the null for each malformation [Table 2; (43, 52)]. Specifically, Van Gelder et al. reported associations close to the null for anotia/microtia (OR: 0.9, 95% CI: 0.5–1.7), cleft lip with or without cleft palate (OR: 1.0, 95% CI: 0.8–1.3), and cleft palate [OR: 1.0, 95% CI: 0.7–1.5; (52)]; and Bandoli et al. reported an association close to the null for oral cleft [RR: 1.1, 95% CI: 0.9, 1.5; (43)].

## 4. Discussion

This systematic review found mixed and inconclusive associations between *in utero* cannabis exposure and risk for structural birth defects. Results were mixed among (a) the four articles reporting on adjusted associations with cardiac malformations (9, 43, 45, 52), (b) the three articles reporting on adjusted associations with central nervous system malformations (43, 50, 52), and (c) the three articles reporting on adjusted associations with gastrointestinal malformations (43, 51, 52). Some studies suggested *in utero* cannabis exposure was not associated with these types of birth defects; and, other articles suggesting that *in utero* cannabis exposure was associated with increased risk of these types of birth defects. Only two articles reported on adjusted associations with orofacial malformations (43, 52); and, only single articles reported on adjusted associations with eye malformation (43), genitourinary malformations (52), and musculoskeletal malformations (52). Though the articles reporting on associations with orofacial, eye, genitourinary, and musculoskeletal malformations all suggested that *in utero* cannabis exposure was not associated with these types of malformations (43, 52), strong conclusions cannot be drawn from these few studies that all had limitations.

There were several limitations of the included studies that may have contributed to the mixed findings on *in utero* cannabis exposure and birth defects. These limitations are similar to those of studies on *in utero* cannabis exposure and other outcomes, such as long-term neurodevelopmental and psychiatric problems (15). First, many of the studies had samples that were relatively small (e.g., 6 of the 20 studies had samples under 500) and reported findings with wide confidence intervals. Therefore, these studies had poor precision and likely were underpowered to detect associations that truly exist. Second, several studies (i.e., 16 of 20) utilized birth cohorts with births occurring more than 20 years ago, which could be problematic given increasing cannabis potency in recent years (55–57) and the proliferation of newer modes of administration (e.g., vaping, edibles) with potentially different risk profiles (58). Third, most studies utilized self-report data, which may underestimate cannabis exposure (59, 60). Therefore, these studies may have mistakenly classified exposed offspring as unexposed, reducing the likelihood of detecting a true association. Fourth, many studies did not address timing of exposure, which is particularly problematic when studying birth defects given that exposures early in pregnancy may be particularly risky for the development of major structural birth defects (61). Fifth, most studies did not assess associations with dose of cannabis exposure. This is a major limitation given that some research has supported a dose-response relationship between *in utero* cannabis exposure and birth defects (9), and research has shown dose-dependent associations between *in utero* cannabis exposure and other outcomes (8). Sixth, most studies did not adequately account for potential confounders, such as co-exposure to other substances. Despite the high co-occurrence of cannabis use and use of other substances, particularly tobacco and alcohol, in pregnancy 12 of the 20 studies did not take this into consideration. Therefore, observed associations between *in utero* cannabis exposure and birth defects could be attributable to exposure to a substance other than cannabis or could be explained by an interactive effect of cannabis use plus use of another substance (62, 63). We note that one study did find similar associations with and without limiting the sample to pregnancies with substance use disorder diagnoses other than cannabis-related diagnoses (43). Nonetheless, more research is needed to parse apart the effects of *in utero* cannabis exposure from exposure to other substances.

It is important to recognize that the mixed and inconclusive results on associations between *in utero* cannabis exposure and structural birth defects should not be interpreted as evidence suggesting cannabis use in pregnancy is safe. Rather these results indicate that the relationship between *in utero* cannabis exposure and structural birth defects is unknown and point to a critical need for future research. This need is particularly pressing given the documented increasing rates in prenatal cannabis use (1).

There are several important avenues for future research. First, samples should be sufficiently large to have adequate statistical power to identify associations if they truly exist. Second, studies with large sample sizes should evaluate associations with specific malformations within organ-specific malformation groups. Third, studies would benefit from including samples comprised of recent birth cohorts given changes in cannabis potency and modes of administration that have occurred in recent years. Fourth, utilizing biological measures (e.g., urine toxicology tests) in addition to self-reported cannabis use would reduce measurement error related to *in utero* cannabis exposure. Fifth, assessing the influence of timing of exposure and particularly focusing on first-trimester exposure is important. Sixth, it is also important for future studies to quantify the amount of prenatal cannabis exposure by considering the dose, frequency, potency, mode of administration and duration of use during pregnancy. Seventh, studies should utilize methods that rigorously evaluate the potential influence of confounding factors. Using conceptual models based on previous literature, researchers can identify potential factors that may confound associations between *in utero* cannabis exposure and birth defects. Researchers could also consider using advanced epidemiological methods that have been utilized to study other *in utero* exposures to help adjust for confounding factors, such as propensity scores, cannabis use before but not during pregnancy as a comparator, and comparisons of differentially exposed siblings [see Sujan et al. for a review of methods that have been used to study antidepressant medications during pregnancy (64)].

Importantly, no single study can implement all of these recommendations, particularly given common obstacles faced by researchers, such as funding limitations restricting the scope of studies, challenges enrolling participants, difficulty obtaining biological samples, and loss to follow-up. However, future research should try to incorporate as many of these recommendations as possible to reduce biases and maximize the overall quality of the studies. Rigorous, high-quality information on the potential consequences of *in utero* cannabis exposure is vital for individuals to make informed choices about cannabis use in pregnancy, as well as for families and providers caring for infants exposed to cannabis *in utero*.

## Data Availability

The original contributions presented in the study are included in the article. Further inquiries can be directed to the corresponding author.
